# Heat and Infant Mortality

**Published:** 1914-05

**Authors:** 


**Heat and Infant Mortality,** by J. W. Schereschewsky, Surgeon, United States Public Health Service. Reprint No. 155 from the Public Health Reports, Dec. 5, 1913.

1. The action of heat as a direct cause in the summer mortality of infants has been greatly underestimated in the last 25 years. In the future much more weight should be given to its influence.

2. The lethal action of heat is a function, not so much of the maximum and mean temperatures of the external air as of the indoor temperatures, which, in the late summer, may continue to be high, in spite of remissions in temperature of the external air.

3. The action of dirty and stale milk in causing the death of infants has been given a significance which has overshadowed other factors of equal or greater importance.

4. There is evidence to show that a certain proportion of infant deaths are due to specific infections, in the dissemin ation of which contact infection and flies doubtless play a part.

5. As a result, future activities for the prevention of infant mortality must concentrate themselves to a greater extent on the question of housing, especially the conditions productive of high indoor temperatures, such as overcrowding, narrow streets, and the absence of through ventilation.

6. Poor housing conditions can be partially neutralized by the proper care of babies in the summer. The general public should be educated as to the importance of high indoor temperature in causing infant diseases.

